# Standardized gene nomenclature for the *Brassica *genus

**DOI:** 10.1186/1746-4811-4-10

**Published:** 2008-05-20

**Authors:** Lars Østergaard, Graham J King

**Affiliations:** 1 Department of Crop Genetics, John Innes Centre, Norwich, NR4 7UH, UK; 2 Plant Science Department, Rothamsted Research, Harpenden, AL5 2JQ, UK

## Abstract

The genus *Brassica *(Brassicaceae, Brassiceae) is closely related to the model plant *Arabidopsis*, and includes several important crop plants. Against the background of ongoing genome sequencing, and in line with efforts to standardize and simplify description of genetic entities, we propose a standard systematic gene nomenclature system for the *Brassica *genus. This is based upon concatenating abbreviated categories, where these are listed in descending order of significance from left to right (i.e. genus – species – genome – gene name – locus – allele). Indicative examples are provided, and the considerations and recommendations for use are discussed, including outlining the relationship with functionally well-characterized *Arabidopsis *orthologues. A *Brassica *Gene Registry has been established under the auspices of the Multinational *Brassica *Genome Project that will enable management of gene names within the research community, and includes provisional allocation of standard names to genes previously described in the literature or in sequence repositories. The proposed standardization of *Brassica *gene nomenclature has been distributed to editors of plant and genetics journals and curators of sequence repositories, so that it can be adopted universally.

## Introduction

**Figure 1:**
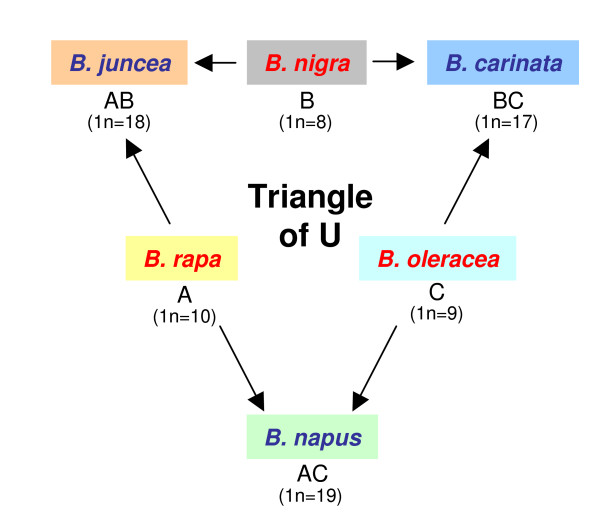
The genetic relationship between *Brassica *species of the "Triangle of U" [1]. Diploid species are indicated by red font, allotetraploid (amphidiploid) species by blue. The background shading for species text boxes is for ease of identification and is also adopted within the *Brassica *gene registry [5] and *Brassica *reference chromosome assignment site [3]. Cytodemes are indicated below the species names, with the number of chromosomes in the haploid genomes indicated in parenthesis.

The genus *Brassica *(Brassicaceae, Brassiceae) is closely related to the model plant *Arabidopsis*, and includes several important crop plants such as oilseed rape (Canola), brown mustard, Chinese cabbage, turnip, cabbage, cauliflower and broccoli. In nature the three diploid *Brassica *species forming the "Triangle of U" [1]* B. rapa*, *B. nigra *and *B. oleracea *have hybridized in all possible combinations to produce the three allotetraploid species *B. juncea*, *B. napus *and *B. carinata *(Figure 1). The genomes of *B. rapa*, *B. nigra *and *B. oleracea *have been named A, B and C, respectively. Therefore the resulting amphidiploid cytodemes become AB, AC and BC for *B. juncea*, *B. napus *and *B. carinata*, respectively. As well as the canonical diploid species, current taxonomies describe a number of additional, non-domesticated, species, with at least ten described within the C genome cytodeme. Although different authors have allocated distinct nomenclature to genes and paralogues identified in *Brassica*, at present there is no agreed convention for assigning names. This has already resulted in instances of both synonyms and homonyms in the literature and sequence repositories (*e. g*. Genbank). We anticipate that this situation will be greatly exacerbated over the next few years as large numbers of sequences are acquired, characterized and annotated.

A multinational project to sequence the *Brassica *A genome has been initiated. The participating partners are using a BAC-by-BAC approach to sequence the gene space of *B. rapa *to Phase 2 quality sequence [2]. This will be invaluable in combination with future and ongoing *Brassica *genomics initiatives such as TILLING populations, genome-wide expression studies and integration of linkage maps. There is, however, a danger that the challenge of dealing with this amount of data prevents effective use of the information. In order to avoid unnecessary inconsistencies, confusion and complexity, it is important that scientists can communicate as simply and comprehensibly as possible. The best way to achieve this is to use a common systematic (genetic) language when referring to genotypes, accessions, gene names etc. This exercise in formalization of *Brassica *gene names should not be confused with requirements for systematic annotation of genome sequence, where gene models are usually ascribed arbitrary codes with no functional semantic content apart from that which refers to accession or location within the genome.

### Proposed systematic gene nomenclature for the *Brassica *genus

As a result of the increasing convergence of information arising from the ability to align linkage maps, chromosomes and genomic sequences, the *Brassica *research community has recently agreed to assign consistent chromosome/linkage group nomenclature to the three diploid *Brassica *genomes, and to use this when referring to linkage groups in the amphidiploid species [3].

In line with this move towards standardization and simplification, we propose a standard systematic gene nomenclature system for the *Brassica *genus where categories are listed in descending order of significance from left to right (i.e. genus – species – genome – gene name – locus – allele). The syntax proposed is of the form:

<GENUS 1 LETTER> [<species 2 letters>]<GENOME 1 LETTER>|<X>.<NAME 3–6 LETTER CODE>.<locus assignment 1 letter>

where < > surrounds categories, [] indicates an optional item and | denotes "or". When referring to gene names, the string is italicized, whilst the corresponding protein name is not.

For example, an expected orthologue of the *Arabidopsis INDEHISCENT *(*IND*) gene [4] isolated from the A genome of *B. napus *would be assigned:

BnaA.IND.a

Further examples are shown in Table 1 and in the gene registry [5]. When preparing a manuscript for publication, we recommend that authors use the systematic name on the first occasion that the gene is mentioned. For clarity, however, it may be favourable to use a shortened synonym throughout the remainder of the paper, and this should be at the discretion of the authors and journal editors.

**Table 1:** Examples indicating use of the proposed systematic *Brassica *gene nomenclature system.

**Example**	**Description**	**Comment**
*BraA.IND.a*	Locus 'a' paraloque of the *IND *gene in the A genome species *Brassica rapa*	-
*BnaC.IND.b*	Locus 'b' paraloque of the *IND *gene on the C genome of *B. napus*	-
*BjuX.IND.a*	Locus 'a' paraloque of the *IND *gene in *B. juncea*. Genome unknown	Provisional name until more assigned to specific genome
*BraA.IND.a1*	Allele 1 at locus a of the *IND *gene on the A genome of *B. rapa*	-
*BniB.IND.b*	Locus 'b' paraloque of the *IND *gene on the B genome of *B. nigra*	Does not necessarily refer to homeologous locus of *BnaC.IND.b*
*braA.ind.a-1*	Mutant allele of *BraA.IND.a*	Mutant annotation in accordance with system used in *Arabidopsis *[11]
*BinC.IND.a*	Locus 'a' paralogue of the *IND *gene in the C genome species *B. insularis*	-
*BolC.SP11.a-64*	Allele number 64 of locus 'a' of the *SP11 *gene of *B. oleracea*	Notice that the family member number is included in the gene name category

### Considerations

**1**. Adopting two letters to indicate species, rather than one letter, is consistent with the standard nomenclature recently developed to describe *Brassica *linkage maps [3], and is designed to reduce ambiguity between, for example, *B. napus *(Bna) and *B. nigra *(Bni). In some situations it is reasonable to argue that it is unnecessary to indicate the species name altogether, and therefore we retain this as an option. However, there are likely to be circumstances during the period prior to availability of full contiguous sequence of each genome, where this additional clarity is required. For example, if the gene has been isolated from an amphidiploid and the genome is unknown (indicated by X – see below, and Table 1), it is not immediately obvious from which species the gene has been isolated. There are additional benefits from the explicit assignment of species, especially in assisting comprehension for non-*Brassica *specialists. In any case, we leave this to individuals whether to include this or not.

**2**. Use of the letter "X" in place of a specific genome assignment indicates that the genome of origin is currently unknown or ambiguous (Table 1). This would be appropriate, for example, when a gene is isolated from an amphidiploid species, but has yet to be mapped unequivocally to a specific genome. It is expected that the name would be updated when the relevant information becomes available. The proposed syntax does not include chromosome number assignment, as this is outside the scope of gene nomenclature prior to full genome annotation. Definitive chromosome number assignment, orientation and map integration within the *Brassica *triangle of U is due to be reported in full in a forthcoming publication [3].

**3**. The "NAME" category is expected to be based either on the name of an orthologous gene previously identified in another organism, or a novel appellation. The numbering of individual gene family members should also be included in this category (Table 1). Since the *Brassica *genus is closely related to that of *Arabidopsis thaliana *priority will be given to orthologues from *Arabidopsis *rather than more distantly related species. For example, a *Brassica oleracea *orthologue of the *APETALA1 *(*Arabidopsis*) gene, the orthologue of which was originally identified in *Antirrhinum *as *SQUAMOSA *[6,7], should be named *BolC.AP1.a *rather than *BolC.SQUA.a*.

**4**. Since most genes are expected to exist as multiple (≥ 2–3) copies in *Brassica *diploid genomes [8-10], it will be important to distinguish between these paralogues. This has already been addressed by several authors by assigning suffixes to each locus. We therefore propose to extend and formalize this by allocating a lower-case letter as a suffix according to an accession policy – where the first identified locus would be assigned as *a*, the second as *b*, *et seq *(Table 1). In situations where more than one allele has been described for the same locus, we suggest an additional integer following the locus identifier.

A full stop/period ('.') is introduced prior to the locus letter to separate gene name and locus when dealing with mutants where genus and gene name categories are also written in lower case letters (Table 1).

We do not expect that it will be possible for some time that locus assignments will be able to be directly compared across genomes, since this would require that the sequence of all paralogues from all genomes be available. Once complete contiguous genome sequences become available, an inventory of ordered annotated gene models is expected to be assigned and described retrospectively in terms of any extant genes, as has been the case with, for example, *Arabidopsis*. Therefore it should not be assumed that it is necessarily the case that *BnaC.IND.b *on the C genome and *BniB.IND.b *on the B genome refer to homoeologous loci (Table 1).

For *Brassica *lines that contain mutations within a given gene, the "*genus*" and "*name*" categories will be written in lower case and italicized letters, with the allele designation indicated by a hyphen followed by a number, as is the standard for other species such as *Arabidopsis thaliana *[11] (Table 1). It should be noted that by "mutation" we refer to chemically or physically induced alterations in the DNA sequence, but the allele designation described here can also be extended to naturally occurring alleles that may or may not have altered function compared to 'wild types'.

In conclusion, the system proposed here is consistent with existing initiatives and accepted practice for standardizing gene nomenclature in other genera [12], and should thus be easily comprehensible to scientists outside the *Brassica *research community.

A *Brassica *Gene Registry for management of gene names has been established [5], and we urge the research community to check this web page and use it to register gene names. Applying the rules described here, we have allocated provisional new names to *Brassica *genes that have already been described in the literature or in sequence repositories. These can be searched based upon their original synonyms, Genbank accession, GI number or other classifiers. Decisions on allocation of names where homonyms may arise will be discussed amongst members of the Multinational *Brassica *Genome Project Steering Committee.

Where the function of a gene is elucidated through *eg*. forward genetic screens and subsequent cloning, the naming of the gene is conventionally based on a characteristic developmental defect apparent in the mutant. We do not wish to discourage this naming procedure for *Brassica *genes. However, we anticipate that when initially described, gene names will be constructed according to the systematic syntax described above, and that this will be allocated at the time of first use in the associated original publication. It is recommended that at this time both the systematic name and the descriptive name be submitted to the *Brassica *gene registry [5].

## Conclusion

We propose a standardized system for gene nomenclature in the genus *Brassica *to facilitate communication among scientists in the *Brassica *research community and to make the field easily accessible for non-*Brassica *researchers.

The proposed standardization of *Brassica *gene nomenclature has been distributed to editors of plant and genetics journals and to Genbank and EMBL so that it can be immediately implemented in the literature. We hope that this will assist the community in reaching a consensus terminology, provide clarity and thus facilitate scientific communication and data integration.

## Authors' contributions

LØ and GJK wrote the manuscript together, and GJK established the gene registry. Both authors read and approved the final manuscript.
